# Bone matrix components activate the NLRP3 inflammasome and promote osteoclast differentiation

**DOI:** 10.1038/s41598-017-07014-0

**Published:** 2017-07-26

**Authors:** Yael Alippe, Chun Wang, Biancamaria Ricci, Jianqiu Xiao, Chao Qu, Wei Zou, Deborah V. Novack, Yousef Abu-Amer, Roberto Civitelli, Gabriel Mbalaviele

**Affiliations:** 1 Division of Bone and Mineral Diseases, Washington University School of Medicine, St. Louis, MO 63110 United States; 2 Department of Orthopedic Surgery, Washington University School of Medicine, St. Louis, MO 63110 United States; 3 Department of Pathology and Immunology, Washington University School of Medicine, St. Louis, MO 63110 United States

## Abstract

The NLRP3 inflammasome senses a variety of signals referred to as danger associated molecular patterns (DAMPs), including those triggered by crystalline particulates or degradation products of extracellular matrix. Since some DAMPs confer tissue-specific activation of the inflammasomes, we tested the hypothesis that bone matrix components function as DAMPs for the NLRP3 inflammasome and regulate osteoclast differentiation. Indeed, bone particles cause exuberant osteoclastogenesis in the presence of RANKL, a response that correlates with NLRP3 abundance and the state of inflammasome activation. To determine the relevance of these findings to bone homeostasis, we studied the impact of *Nlrp3* deficiency on bone using pre-clinical mouse models of high bone turnover, including estrogen deficiency and sustained exposure to parathyroid hormone or RANKL. Despite comparable baseline indices of bone mass, bone loss caused by hormonal or RANKL perturbations is significantly reduced in *Nlrp3* deficient than in wild type mice. Consistent with the notion that osteolysis releases DAMPs from bone matrix, pharmacologic inhibition of bone resorption by zoledronate attenuates inflammasome activation in mice. Thus, signals originating from bone matrix activate the NLRP3 inflammasome in the osteoclast lineage, and may represent a bone-restricted positive feedback mechanism that amplifies bone resorption in pathologic conditions of accelerated bone turnover.

## Introduction

Pathological conditions such as estrogen deficiency and hyperparathyroidism cause high bone turnover, and ultimately, a net bone loss, as a result of bone resorption overcoming bone formation over time. Chronic low grade inflammation attended by cytokines, including IL-1β and TNF-α, has been linked to bone loss associated with estrogen insufficiency^1–3^. Persistent excessive production of parathyroid hormone (PTH) also causes bone loss through mechanisms involving up-regulation of receptor activator of NF-κB ligand (RANKL) expression, the obligatory factor for osteoclast-mediated bone resorption^4, 5^. Indeed, RANKL administration causes massive bone resorption^6^, consistent with the notion that RANKL abundance drives pathological osteolysis. Thus, accelerated bone resorption can occur in the absence of high grade inflammation, but the signals that sustain such abnormal bone resorption are not known.

The current dogma on bone resorption posits that osteoclasts (OC) acidify the resorption lacuna, resulting in the dissolution of the inorganic components of the bone extracellular matrix, including hydroxyapatite^7^. This reaction exposes the organic phase of the bone matrix, which is then degraded by secreted lysosomal enzymes, mainly the cysteine protease, cathepsin K^7^. Evidence also indicates that both the organic and inorganic degradation products from bone matrix are endocytosed via the OC ruffled membrane^8–10^. This process enables OC to excrete degraded matrix components while digging deep into bone and maintaining an enclosed resorption site. Consistent with this concept, collagen I degradation products such as C-telopeptide of type I collagen are found in biological fluids and are used as markers of bone resorption^11^. Thus, bone degradation products should in theory interact with the OC lineage, but whether these materials function as danger-associated molecular patterns (DAMPs) and activate the inflammasomes in these cells is not known.

The inflammasomes are intracellular protein complexes expressed mainly by myeloid cells from which the osteoclasts arise^12^. They are assembled by various receptors, including nucleotide-binding oligomerization domain, leucine-rich repeat-containing proteins (NLRP1, NLRP3, NLRP6 and NLRP12), absent in melanoma 2 (AIM 2)-like receptors (ALRs) or pyrin^13^. These receptors recognize microbial structures known as pathogen-associated molecular patterns (PAMPs), and participate in the restoration of tissue integrity after injury upon sensing the debris from damaged cells, signals known as danger-associated molecular patterns (DAMPs)^13–15^. Ligand recognition or sensing leads to sequential recruitment of apoptosis-associated speck-like protein containing a CARD (ASC) and pro-caspase-1, which is then converted into active caspase-1^16, 17^. Activated inflammasomes are involved primarily in the conversion of pro-IL-1β and pro-IL-18 into biologically active, IL-1β and IL-18, respectively^13^.

The NLRP3 inflammasome is implicated not only in inflammatory disorders^18^, but also in numerous metabolic diseases driven by low grade inflammation, some of which are caused by specific endogenous components. Indeed, the NLRP3 inflammasome is activated by various host DAMPs such as glucose in type-2 diabetes, cholesterol crystals in atherosclerosis and fatty acid in obesity^14^. More to the point, it was reported recently that loss of NLRP3 attenuates osteopenia associated with aging in mice, though the underlying cellular mechanisms were not studied in detail^19^. Thus, whether the NLRP3 inflammasome plays an important role in bone resorption in conditions of low grade inflammation, and whether bone matrix components participate in this process remains largely unknown.

In this study, we find that bone matrix components promote osteoclastogenesis robustly in WT, but to a lesser extent in *Nlrp3* ^−/−^ cells. Importantly, osteopenia caused by high bone turnover is reduced significantly in mutant mice compared to WT mice. Moreover, pharmacologic inhibition of bone resorption by zoledronate correlates with attenuated inflammasome activation *in vivo*, suggesting that bone resorption releases the activators of the inflammasomes and stimulators of OC differentiation from bone matrix. Thus, the bone DAMPs-NLRP3 inflammasome axis may represent a novel mechanism that sustains bone resorption.

## Results

### Bone matrix components stimulate the expression of inflammasome constituents

**Figure 1:**
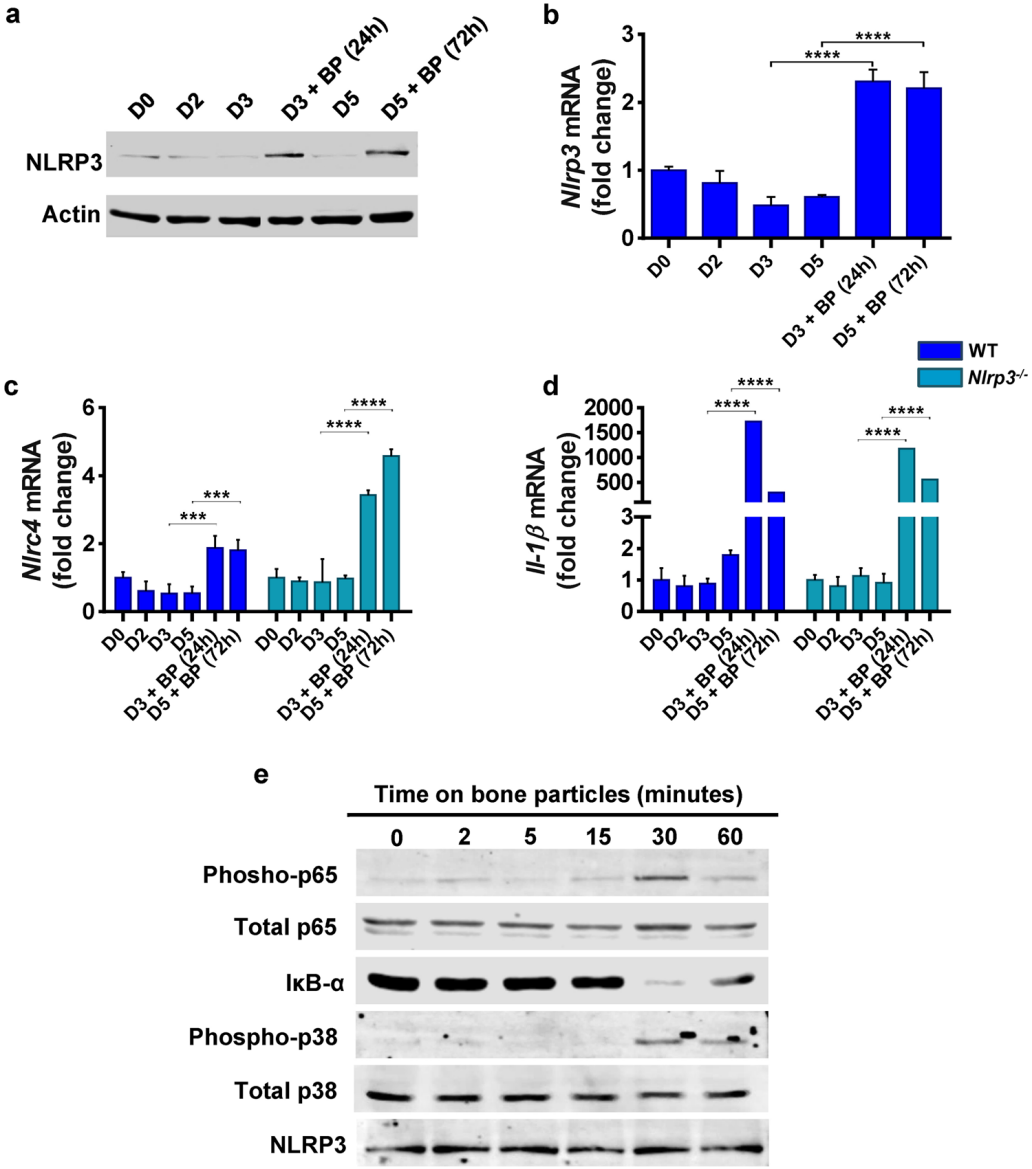
Bone matrix components stimulate the expression of inflammasome constituents. BMM from WT or *Nlrp3* ^−/−^ mice were incubated with 50 ng/ml RANKL in the presence of CMG as a source of M-CSF. Cells pre-treated with RANKL for 2days (D2) were exposed to bone particles (BP, 125 µg/ml) or PBS for 24 hours or 72 hours (h). Cells were harvested on D0 (the first day of RANKL stimulation), D2, D3 or D5 and protein and mRNA expression was assessed. (**a**) Western blot analysis of NLRP3 expression. Data are representative of at least 3 independent experiments. Results are from the same gels, but the lanes were cut and pasted (see S1f). (**b**–**d**) qPCR analysis of mRNA expression. Data are expressed as means ± SD, and are representative of at least 3 independent experiments. (**b**) ****p < 0.0001 (one-way ANOVA and Sidak’s multiple comparison test); (**c**) WT: ***p < 0.0005, *Nlrp3* ^−/−^: ****p < 0.0001; (**d**) ****p < 0.0001. (**e**) Western blot analysis of pathway activation. WT BMM were serum-starved overnight and stimulated with 125 µg/ml BP. Cells were harvested at the indicated time points.

Crystalline and organic materials are potent activators of the inflammasomes^20–23^, both of which are released from bone extracellular matrix (ECM) during bone resorption, and traffic through bone-resorbing OC^8–10^. We tested the hypothesis that bone degradation products function as DAMPs and regulate the abundance and/or activity of the inflammasomes in the OC lineage. The expression of NLRP3 (Fig. 1a and b), *Nlrc4* (Fig. 1c) and several other components of the inflammasomes (e.g., *Nlrp1*, *Aim*2, *Asc*, *caspase-1* or *Il-18*; Fig. S1) if anything, decreased slightly during RANKL-induced OC differentiation of WT or *Nlrp3* ^−/−^ bone marrow macrophages (BMM). In contrast, exposure of committed OC precursors (BMM treated with RANKL for 2 days) to bone particles, which were prepared by shredding bovine cortical bones, increased *Nlrp3* mRNA and protein expression in WT cells (Fig. 1a and b). Bone particles also stimulated *Nlrc4* (Fig. 1c) and *Il-1β* (Fig. 1d) mRNA expression in WT and *Nlrp3* ^−/−^ cells whereas the abundance of the other inflammasome elements was in general unaffected (Fig. S1a–e). To gain insight into the mechanisms through which bone particles regulate the expression of inflammasome components, we studied the activation of NF-κB and p38 MAPK, pathways that stimulate the expression of pro-inflammatory cytokines^24^. Phosphorylation of the NF-κB subunit, p65, and p38 MAPK was induced by bone particles (Fig. 1e). Phosphorylation of p65 inversely correlated with the abundance of the NF-κB inhibitor, IκBα, as expected (Fig. 1e). These results are consistent with the current paradigm that NF-κB is the indispensable transducer of the so-called priming signals necessary for pro-IL-1β and NLRP3 induction^25, 26^.

**Figure 2:**
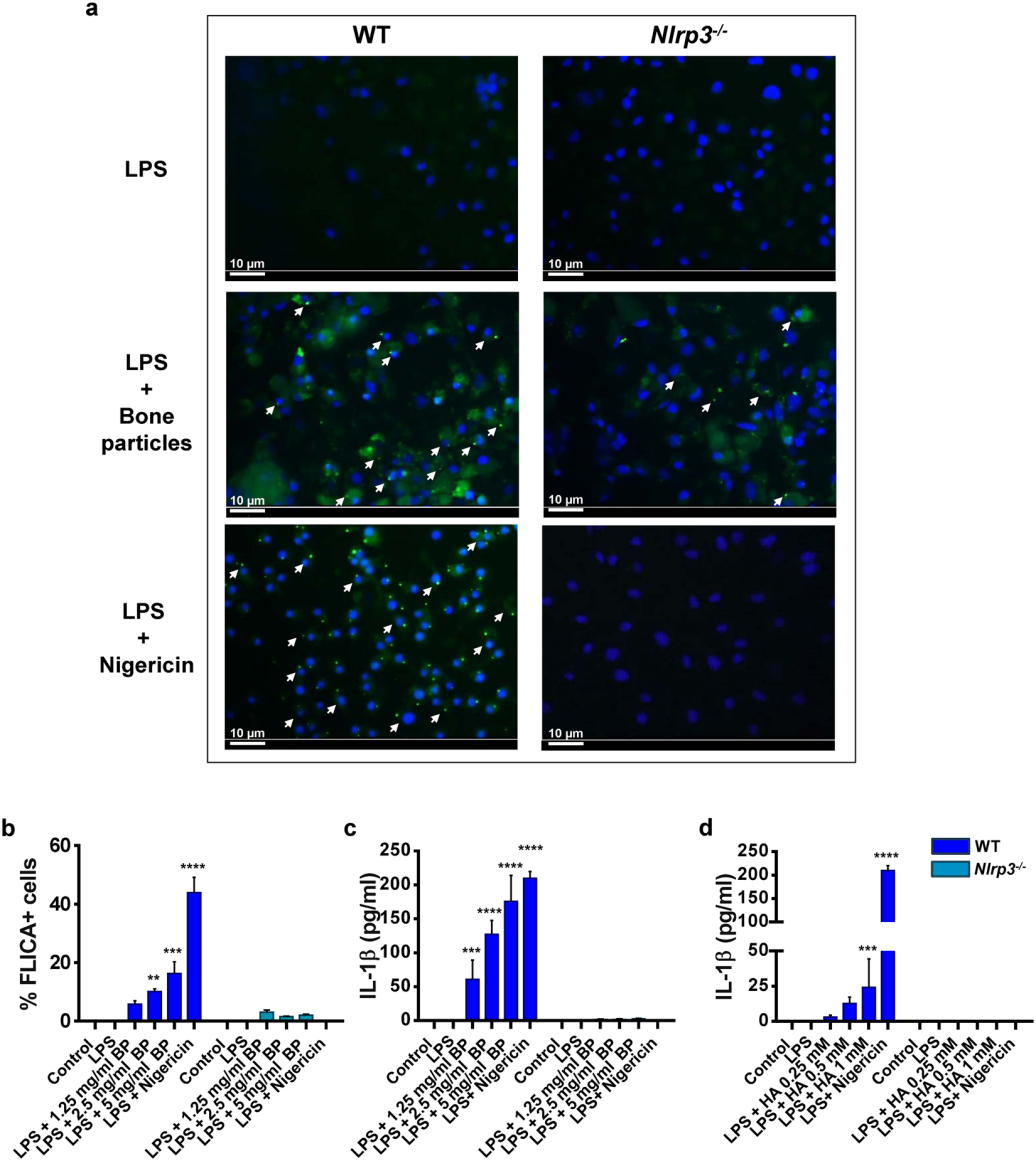
Bone matrix components provide secondary signals that assemble functional inflammasomes. WT or *Nlrp3* ^−/−^ BMM were incubated for 3 hours with 100 ng/ml LPS and stimulated with 15 µM nigericin for 30 minutes, bone particles (BP, 1.25–5 mg/ml) or hydroxyapatite (HA) crystals (0.25–1 mM) for 1 hour. (**a**) Cells were then incubated with FLICA^TM^ FAM-YVAD-FMK probe and analyzed by fluorescence microscopy. Original magnification, 20x; scale bars = 10 µm; white arrows show foci of caspase-1 activation. (**b**) Quantification of FLICA^+^ cells. Data are means ± SD of at least 3 fields. (**c**,**d**) IL-1β release in the conditioned media. Data are means ± SD from experimental triplicates, and are representative of at least 2 independent experiments. (**b**) **p = 0.0062, ***p = 0.0003, ****p < 0.0001; (**c**) ***p = 0.0008, ****p < 0.0001. p values correspond to significant differences relative to the control for each genotype.

We also determined whether bone particles can provide secondary signals that assemble functional inflammasomes. Inflammasome activation can be monitored by measuring IL-1β secretion or by fluorescence microscopy based-detection of cellular foci upon cell incubation with FLICA^TM^ FAM-YVAD-FMK probe, which binds covalently to active caspase-1. Upon exposure to bone particles, the percentage of cells with active caspase-1 was significantly increased in LPS-primed WT BMM compared to *Nlrp3* ^−/−^ BMM (Fig. 2a and b; green foci, white arrows). The NLRP3 inflammasome activator, nigericin, used as positive control, induced caspase-1 activity in WT, but not *Nlrp3* ^−/−^ BMM as expected (Fig. 2a and b), suggesting that bone particle-dependent residual staining in mutant cells was caspase-1-independent or reflected the activity of other complexes such as the NLRC4 inflammasome. Interestingly, similar to nigericin, bone particles also increased IL-1β secretion in LPS-primed WT BMM, but not *Nlrp3* ^−/−^ BMM (Fig. 2c). The relatively low concentrations of IL-1β reflect the low cell density, which was optimal for the FLICA readout.

Next, we determined whether hydroxyapatite (HA) crystals, the mineral component of bone particles, can stimulate the NLRP3 inflammasome. Exposure of LPS-stimulated BMM to synthetic HA crystals induced the formation of inflammasome foci (data not shown) and increased IL-1β production in NLRP3-dependent manner (Fig. 2d). Collectively, these results indicate that products from bone matrix function as DAMPs and activate the NLRP3 inflammasome.

### Bone matrix components stimulate OC differentiation

**Figure 3:**
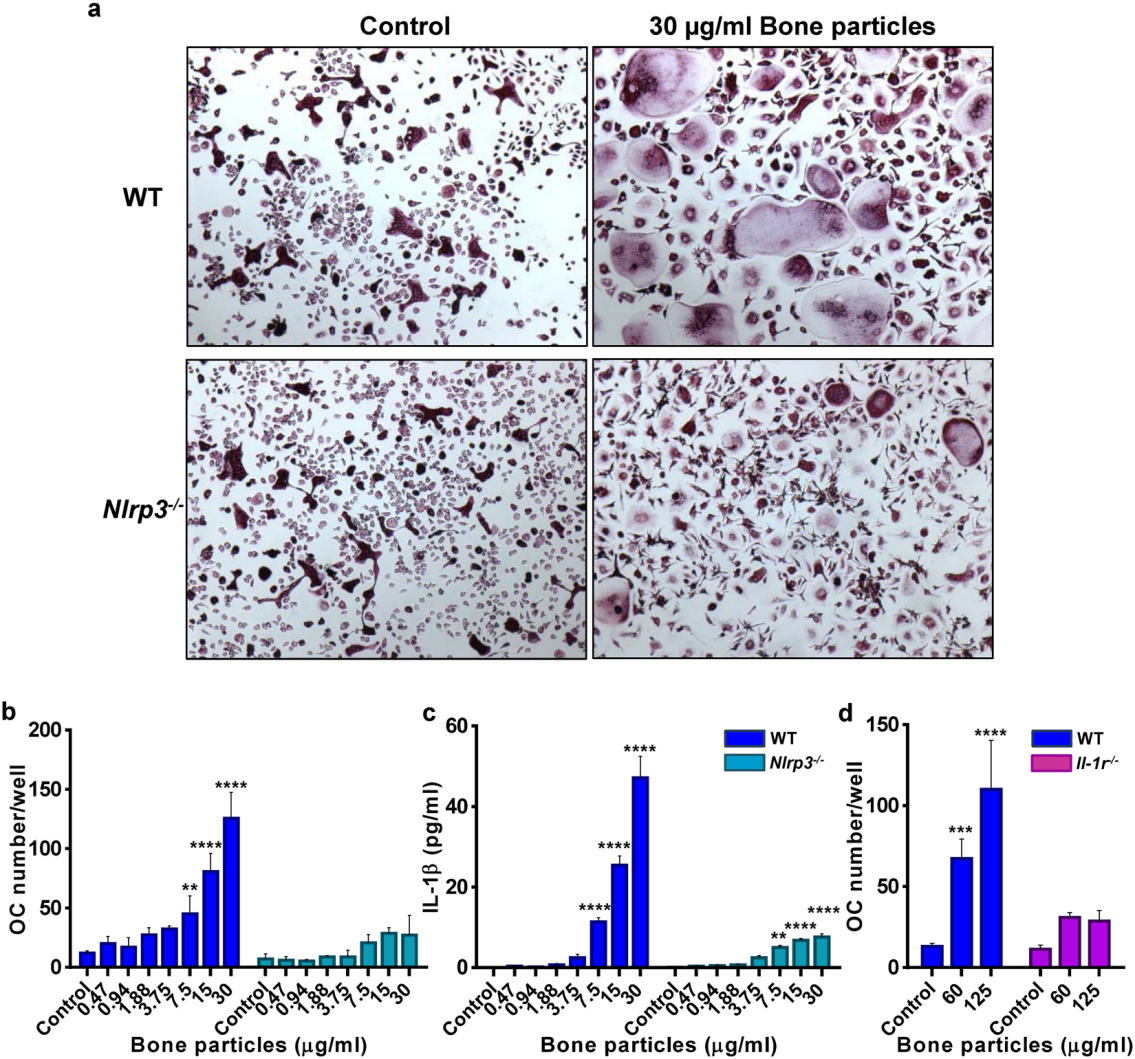
Bone matrix components stimulate OC differentiation. BMM from WT or *Nlrp3* ^−/−^ mice were incubated with 25 ng/ml RANKL for 2 days (D2) then treated with bone particles. (**a**) Cells were stained for TRAP on day 3 (D3). Original magnification, ×2. (**b**) OC number/well. (**c**) IL-1β production was measured in the supernatants from (**b**). (**d**) Effects of IL-1 receptor deficiency on OC formation. Experiments were run in triplicates, and data are means ± SD. (**b**) **p = 0.0013, ****p < 0.0001; (**c**) WT: ****p < 0.0001; *Nlrp3* ^−/−^: **p = 0.0030, ****p < 0.0001; (**d**) ***p = 0.0008; ****p < 0.0001. p values correspond to significant differences relative to the control for each genotype, and data are representative of at least 4 independent experiments.

We hypothesized that bone matrix-derived products may regulate RANKL-dependent osteoclastogenesis through their positive actions on the NLRP3 inflammasome, a known stimulator of OC differentiation and activity^27, 28^. RANKL-induced OC differentiation was not impaired in *Nlrp3* ^−/−^ cells (Fig. 3a and b), consistent with the normal bone mass of mutant mice. Interestingly, bone debris enhanced OC formation, a response that was attenuated in *Nlrp3* ^−/−^ cells (Fig. 3a and b; Fig. S2a–c), and correlated with IL-1β production (Fig. 3c). Low concentration of RANKL (25 ng/ml) and short incubation times (3 days) were used in order to detect synergistic interactions with bone particles. Likewise, HA crystals amplified RANKL actions on OC formation in WT cells, but to a lesser extent in mutant cells (Fig. S3a). Notably, non-mineralized collagen fragments failed to stimulate OC differentiation (Fig. S3b) and activate the NLRP3 inflammasome (data not shown). On the other hand, our studies with demineralized organic phase from bone products were not successful because traces of EDTA, which was used to decalcify bones, remained in the decalcified materials and exhibited cytotoxicity (data not shown).

**Figure 4:**
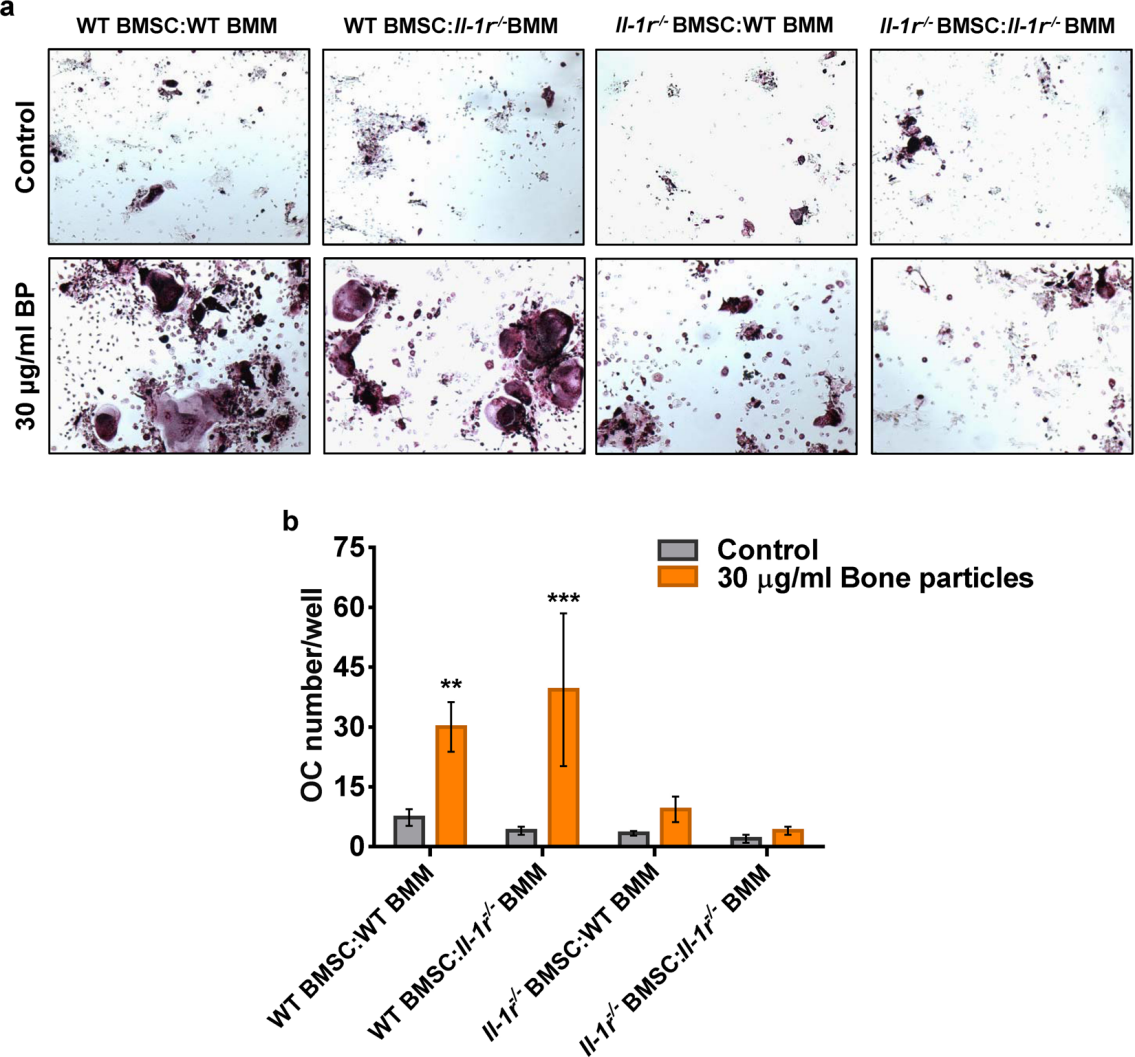
Bone matrix components induce OC formation in co-cultures. BMSC and BMM were obtained from WT or *Il-1r* ^−/−^ mice, and the co-cultures were set up for various cell combinations at a 1:1 ratio. After 2 days of co-cultures, bone particles (BP) were added and TRAP staining was performed at day 4 (**a**), and OC number was determined by counting multinucleated, TRAP-positive cells (**b**). **p = 0.0084, ***p = 0.0004. Data are means ± SD of samples run in triplicates, and are representative of at least 2 independent experiments. p values correspond to significant differences relative to WT BMSC:WT BMM (control).

RANKL acts in concert with TNF-α or IL-1β to regulate osteoclastogenesis^29, 30^. We therefore hypothesized that IL-1β released upon exposure to bone particles may subsequently mediate the osteoclastogenic action of these materials. Consistent with this prediction, while RANKL-driven OC formation was comparable between WT and BMM lacking IL-1 receptor (*Il-1r* ^−/−^), the effects of bone particles on osteoclastogenesis were blunted in mutant cells (Fig. 3d). On the other hand, IL-1β can also affect osteoclastogenesis by up-regulating RANKL and M-CSF expression by bone marrow stromal cells (BMSC)^31^. This raises the possibility that particle-induced IL-1β secretion may enhance OC formation through indirect action of this cytokine on BMSC. We investigated this scenario by carrying out a co-culture system of BMM and BMSC. Bone particles (Fig. 4a; Fig. S4a) or HA crystals (data not shown) induced exuberant osteoclastogenesis, a response that was diminished in BMSC lacking IL-1 receptor. Stimulation of OC formation in WT BMSC and *Il-1r* ^−/−^ BMM co-cultures by bone particles may be mediated by other cytokines such as TNF-α, which was also induced by these materials in BMM cultures (Fig. S4b). Thus, bone DAMPs induce multiple responses, including NLRP3 inflammasome-mediated maturation of IL-1β, which promotes osteoclastogenesis by acting directly on BMM, and indirectly via BMSC.

### Osteopenia associated with high bone turnover is reduced in mice lacking NLRP3

**Figure 5:**
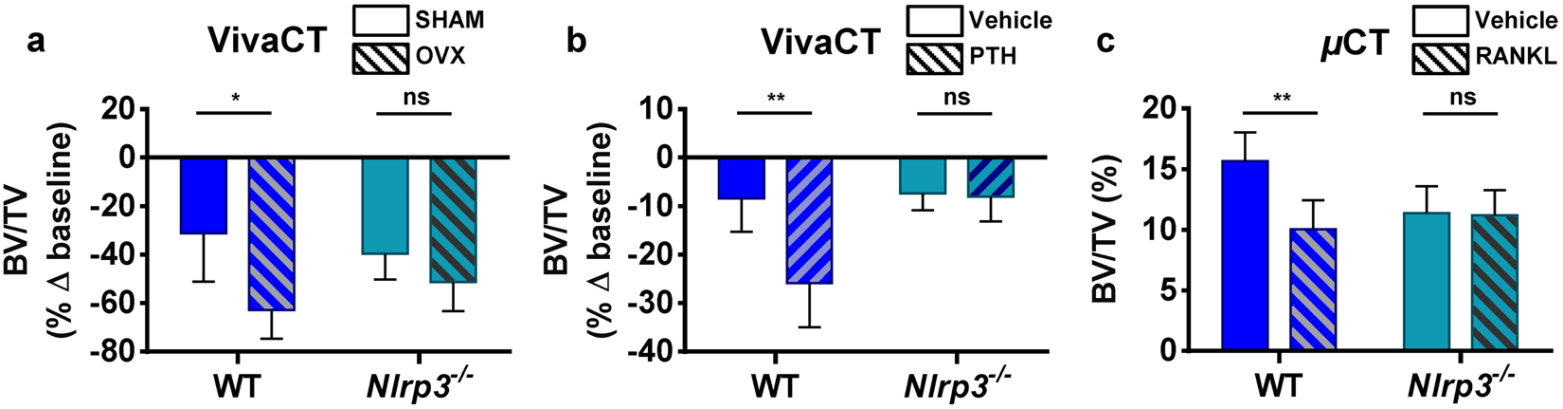
Osteopenia associated with high bone turnover is reduced in mice lacking NLRP3. (**a**) VivaCT analysis was performed on the femurs of 4-month old WT and *Nlrp3* ^−/−^ female mice before and at 8 weeks after OVX or sham surgery. (**b**) VivaCT analysis of 4-month old WT and *Nlrp3* ^−/−^ male mice exposed to PTH (80 μg/kg/day) or vehicle for 2 weeks via osmotic pumps. Data are means ± SD of the percentage of changes relative to baseline (before surgery or treatment) of 4–6 mice/group; (**a**) *p = 0.0111; (**b**) **p = 0.0017. (**c**) Post-mortem µCT scan was performed on the femurs from 3-month old WT and *Nlrp3* ^−/−^ male mice exposed to acute RANKL treatment (1 µg/kg once a day, for 2 days). Data are means ± SD of 4–7 mice/group; **p = 0.0062.

Since IL-1β has been shown to mediate the effects of estrogen deficiency on bone^1–3^, we hypothesized that the NLRP3 inflammasome, via maturation of IL-1β plays a role in estrogen-dependent osteopenia. We therefore studied the effect of ovariectomy (OVX) on bone mass in *Nlrp3* deficiency, a low-grade inflammation model. Baseline femoral bone mass shown as volumetric trabecular bone volume/tissue volume (BV/TV) was comparable between 4-month old WT and *Nlrp3*-deficient female mice based on viva-computed tomography (vivaCT) analysis (data not shown), confirming that NLRP3 is dispensable for bone homeostasis in young adult mice^19^. The analysis performed 8 weeks after surgery revealed significantly reduced BV/TV in WT OVX mice compared to sham-operated mice (Fig. 5a). In contrast, bone parameters were not statistically different between OVX and sham-operated *Nlrp3* ^−/−^ mice (Fig. 5a), suggesting that NLRP3 deficiency protects mice from the excessive bone loss associated with estrogen insufficiency.

PTH stimulates bone resorption via up-regulation of RANKL in osteoblasts and osteocytes^5^. We therefore tested whether the NLRP3 inflammasome is involved in bone resorption driven by PTH. While in WT continuous PTH infusion (80 µg/kg, for 2 weeks) caused 25% loss of BV/TV (~15% larger than in vehicle-treated mice; Fig. 5b), only less than 10% decrease in BV/TV was observed in *Nlrp3* ^−/−^ mice treated with PTH, similar to vehicle-treated groups (Fig. 5b), indicating that lack of *Nlrp3* protects from PTH induced bone loss.

To test whether the NLRP3 inflammasome mediates RANKL effect on bone, mice were given RANKL (1 µg/kg, daily for 2 days) intraperitoneally, an acute regimen that causes substantial bone resorption^6^. As mentioned above, there was no difference in BV/TV between WT and *Nlrp3* ^−/−^ mice at baseline (Fig. 5c). In contrast, RANKL caused approximately 6% loss of BV/TV in WT, but not *Nlrp3*-ablated mice (Fig. 5c). These data are in agreement with the view that the inflammasomes are active mainly in innate immune cells^32^, as RANKL triggers bone resorption through its action on myeloid cell subsets. Thus, despite the inconsistency we noticed related to changes in bone mineral density (BMD), the number (Tb.N) and thickness (Tb.Th) of the trabeculae, and trabecular space (Tb.Sp) (Fig. S5), collectively, our data indicate that the NLRP3 inflammasome promotes bone resorption in various states of accelerated bone turnover such as estrogen deficiency and sustained exposure to PTH or RANKL.

### The inflammasomes are activated in the bone environment in response to signal released during bone resorption

**Figure 6:**
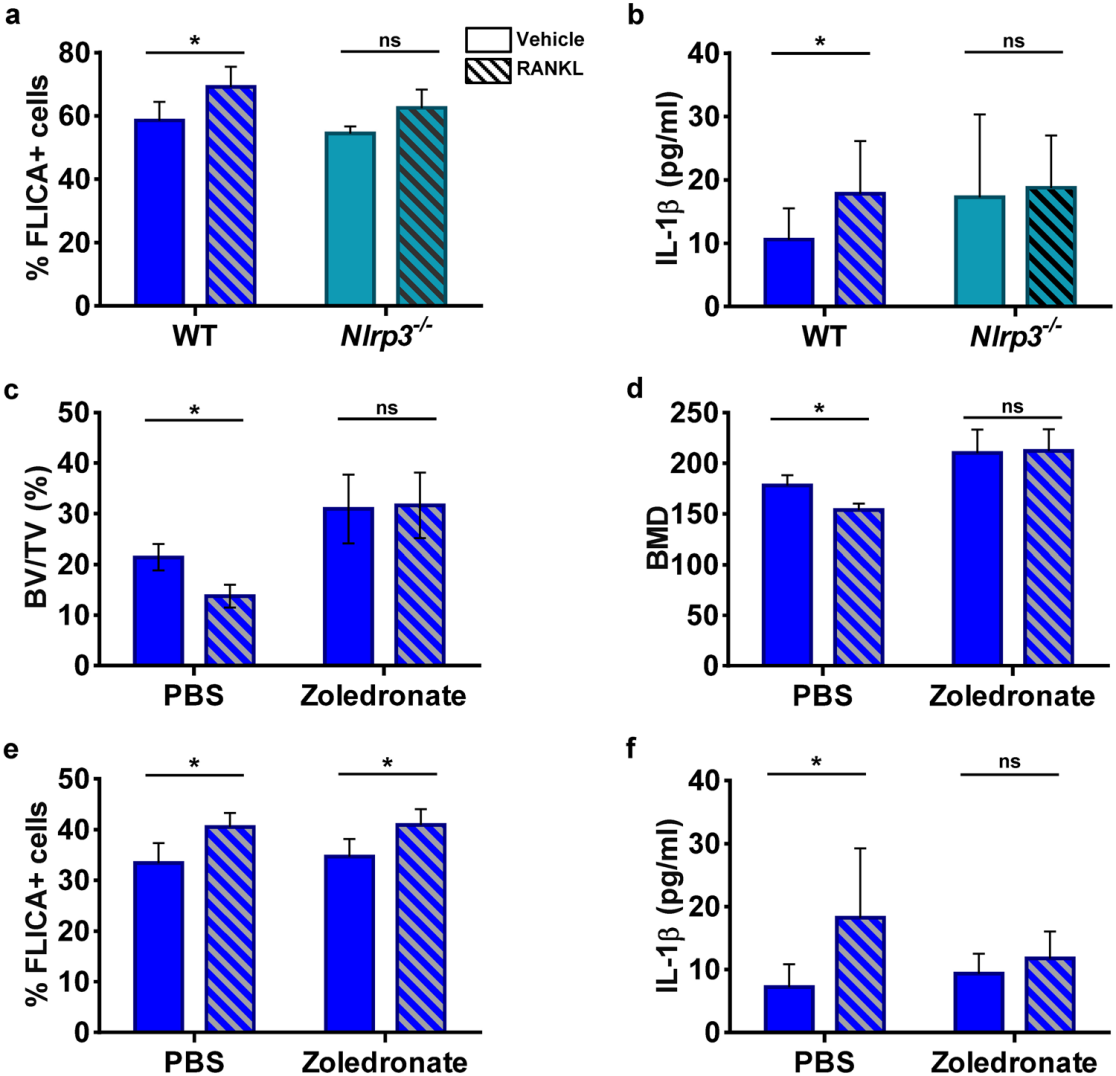
The inflammasomes are activated in the bone environment in response to cues released during bone resorption. RANKL was administrated (1 µg/kg, daily, for 2 days) to WT or *Nlrp3* ^−/−^ 3-month old male mice. (**a**) Percentage of FLICA^+^ cells in whole bone marrow cells determined by flow cytometry (n = 5–7 mice/group); *p = 0.0225. (**b**) IL-1β production in bone marrow (n = 6–16 mice/group); *p = 0.0126 (multiple t-test and Holm-Sidak). (**c** and **d**) WT 3-month old mice were given 40 µg/kg zoledronate or PBS once a week for 4 weeks before RANKL treatment (n = 5 mice/group). Bones were analyzed by µCT. BV/TV: *p = 0.0274; BMD: *p = 0.0351. (**e**) FLICA^+^ bone marrow cells (n = 5 mice/group). *p = 0.0275 for the PBS group; *p = 0.0408 for the zoledronate group. (**f**) IL-1β release in bone marrow (n = 5 mice/group). *p = 0.0286.

The FLICA^TM^ FAM-YVAD-FMK probe can also be used for flow cytometry to assess the state of inflammasome activation^28^. By this method, the percentage of FLICA^+^ mononucleated cells at baseline was similar in 3-month-old WT and *Nlrp3* ^−/−^mice (Fig. 6a). Administration of RANKL increased the percentage of FLICA^+^ cells in WT mice, but not *Nlrp3* ^−/−^ mice (Fig. 6a). RANKL injection also increased IL-1β production in WT (Fig. 6b). In contrast, the cytokine had no noticeable effect on IL-1β secretion in *Nlrp3*-ablated mice (Fig. 6b). It has been recently reported that RANKL induced IL-1β production by the OC lineage^33^. Thus, the inflammasomes including those nucleated by NLRP3 may be involved in bone resorption. We therefore determined whether inhibition of bone resorption by zoledronic acid, a potent bisphosphonate, would alter RANKL-induced bone loss and inflammasome activation in 3-month-old WT male mice. In mice treated with vehicle, RANKL administration induced approximately 10% bone mass loss, and pre-treatment with zoledronate prevented such loss (Fig. 6c and d), results that correlated with microstructural trabecular parameters (Fig. S6). Notably, zoledronate-treated mice had higher bone mass than vehicle-treated animals, also an expected finding (Fig. 6c and d; Fig. S6). Zoledronate had no effects on the percentage of FLICA^+^ cells at baseline or in response to RANKL treatment (Fig. 6e). These results were unexpected, but may be explained by the fact that accurate determinations of intracellular caspase-1 *in vivo* is challenging because caspase-1 is also secreted^25, 34^. We therefore analyzed IL-1β production. We found that zoledronate prevented RANKL-induced increase of IL-1β in bone marrow (Fig. 6f). Taken together with our *in vitro* data showing that bone particles failed to cause IL-1β production in *Nlrp3* ^−/−^ BMM (Fig. 2), we propose that signals released during bone degradation cause inflammasome activation to sustain bone resorption (Fig. S7).

## Discussion

Auto-inflammatory disorders such as neonatal-onset multisystem inflammatory disease (NOMID) are caused by NLRP3-activating mutations, and are associated with osteopenia^35, 36^. The bone outcome is the consequence of chronic inflammatory osteolysis^27^ and is consistent with the central role of this inflammasome in the maturation of IL-1β, a pro-osteoclastogenic cytokine whose levels are markedly elevated in NOMID^27, 37^. While it is established that mutated NLRP3 produces pathogenic effects to bone, little is known regarding the role that WT NLRP3 plays in this tissue. Here, we find that lack of NLRP3 abrogates *in vitro* osteoclastogenesis induced by bone particles, and more importantly, attenuates bone loss in mouse models of post-menopausal osteoporosis and hyperparathyroidism. Others have reported the bone-protective effects of NLRP3-deficiency in age-related osteopenia in mice^19^. Thus, the NLRP3 inflammasome has far-reaching actions in bone homeostasis than originally thought, acting not only in the context of severe inflammation, but also in states of low grade inflammation (e.g., estrogen deficiency or aging). The premise that NLRP3 functions downstream of RANKL signaling is in agreement with our previous work demonstrating that genetic activation of NLRP3 in the OC lineage causes bone resorption in the absence of inflammation^28^. On the other hand, the normal bone phenotype of *Nlrp3* null mice at baseline suggests that other inflammasomes such as those assembled by NLRC4 are also involved in homeostatic bone remodeling and that the NLRP3 inflammasome is more important in pathological osteolytic states.

The NLRP3 inflammasome is activated in response to various stresses, including those triggered by accidental release of lysosomal contents in the cytosol upon rupture of particulate-containing phagolysosomes, excessive levels of mitochondrial-derived reactive oxygen species and K^+^ efflux^12^. Our findings indicating that inflammasome activation in mice is attenuated upon administration of zoledronate imply that this drug inhibits not only bone resorption, but also the release of DAMPs from bone matrix. Potential bone relevant DAMPs include bone degradation products, which have been shown to traffic through the OC, though the impact of the transcytosis process on OC’s function remains unknown. Bone particles and synthetic hydroxyapatite crystals are used as surrogates of bone degradation products and have been shown to modulate numerous bone cell responses, including the differentiation of OC and enhancement of cathepsin K secretion by these cells^38, 39^. Here, we show that bone matrix-derived products also activate the NLRP3 inflammasome and stimulate OC formation. However, the possibility that zoledronate inhibits OC directly through GTPase prenylation^40^ independently of the inflammasome mechanism cannot be ruled out.

Bone particles not only promote the assembly of the inflammasomes, but also stimulate NLRP3, NLRC4 and IL-1β mRNA expression, suggesting that they provide both the priming and secondary signals. Oxidized low-density lipoprotein and hyaluronan have also been shown to generate both types of inputs to the inflammasomes^41–43^. Although we ruled out any contaminations of bone DAMPs by LPS (data not shown), bone debris remain heterogeneous materials comprising mineral and organic components of different sizes and shapes. As a result, elements of these mixtures may affect distinct steps of the inflammasome activation process. However, our findings indicating that hydroxyapatite crystals, but not non-mineralized type 1 collagen fragments activate the NLRP3 inflammasome suggest dominant actions of the mineral phase of bone particles in inflammasome activation. This view is consistent with reports indicating that basic calcium phosphate crystals, which include hydroxyapatite crystals, are potent activators of the NLRP3 inflammasome^44^. Future studies are still needed to determine whether the mineral phase of bone DAMPs acts as particulates or as a source of Ca^2+^. This is important because Ca^2+^ fluxes also activate this inflammasome^45^.

Bone matrix components stimulate IL-1β production in cell cultures, and loss of IL-1 receptor reduces the effects of these particles on osteoclastogenesis, suggesting autocrine and/or paracrine actions of this cytokine in this process. On the other hand, IL-1β release, not the caspase-1 activity readout consistently correlates with NLRP3 inflammasome activation in mice; the reasons for this disparity remain unclear. More to the point, the ability of bone particles to induce IL-1β production and our reliance on IL-1β secretion as readout of inflammasome activation *in vivo* are seemingly at odds with the concept that WT NLRP3 inflammasome can mediate bone resorption in non-inflammatory conditions. We contend that the doses of bone particles or RANKL that we used are exaggerated, but justified in order to inflict cellular perturbations and detect biological changes, including IL-1β secretion. Even if the inflammasome-mediated IL-1β release is enhanced by bone degradation products during bone resorption *in vivo*, such responses likely reflect low grade inflammation tailored to promote bone homeostasis, not to cause disease.

This work introduces a novel concept in bone biology postulating that products originating from bone matrix activate the NLRP3 inflammasome, thus functioning as a positive feedback loop that amplifies bone resorption (Fig. S7). Thus, the bone-protective action of NLRP3 deficiency in various animal models of accelerated bone turnover positions this inflammasome as a target for therapeutic intervention, not only for inflammatory disorders, but also bone metabolic diseases.

## Materials and Methods

### Animals and *in vivo* experiments

*Il-1 receptor* deficient (*Il-1r* ^−/−^) mice and *Nlrp3* null (*Nlrp3* ^−/−^) mice were purchased from Jackson Laboratory. All mice were on C57BL6 background, and mouse genotyping was performed by PCR. To induce estrogen deficiency, bilateral ovariectomy (OVX) or sham surgery (in which ovaries were exteriorized, but replaced intact) were performed under general anesthesia in 4-month old WT or *Nlrp3* ^−/−^ mice, and bones were analyzed 8 weeks after surgery. Estrogen deficiency was confirmed by uterine atrophy. For PTH experiments, 80 μg/kg/day human PTH1-34 or vehicle were delivered to 4-month old WT or *Nlrp3* ^−/−^ male mice for 2 weeks via subcutaneously implanted ALZET osmotic pumps (model-1002, DURECT Corporation) with a delivery rate of 0.25 ml/h as previously described^46^. GST-RANKL or vehicle was administrated intra-peritonally to 3-month old WT or *Nlrp3* ^−/−^ male mice at a dose of 1 µg/kg, daily for 2 days. For pharmacologic inhibition of bone resorption, 40 mg/kg zoledronate (Novartis #484188) were injected subcutaneously to 3-month old WT mice, once a week, for 4 weeks prior to GST-RANKL injection. All procedures were approved by the Institutional Animal Care and Use Committee of Washington University School of Medicine in St. Louis. All experiments were performed in accordance with the relevant guidelines and regulations described in the IACUC-approved protocol #20160245.

### Bone mass and microstructure

Micro-computerized tomography (CT) scanning was performed *in vivo* (Scanco VivaCT40) on each animal before and after surgery or PTH administration as previously described^47^. Post mortem micro-computed tomography (µCT) system (Scanco uCT40) was performed for the evaluation of bone parameters 2 days after GST-RANKL administration. Briefly, femora from mice were stabilized in 2% agarose gel, and µCT scans were taken along the length of the femur as previously described^48^.

### Preparation of bone particles

Bone particles were prepared by shredding bovine cortical bones with an automatic low speed saw (Buehler IsoMet). Particles were washed with 70% ethanol, resuspended in PBS, autoclaved, and washed 3 more times with PBS before the weight of dried bone particles was determined. Hydroxyapatite (HA) crystals were purchased from Sigma (677418, particle size <200 nm) and stock solutions in PBS were exposed to UV light before use. Collagen I was purchased from Santa Cruz Biotechnology (sc-29009), sonicated 2 times for 5 seconds each with the amplitude set at 40 (Sonics Vibracell VC130BP) and filtered with a 0.22 µm syringe filter.

### Cell isolation and cultures

Bone marrow (BM) cells were eluted from femurs and tibias as previously described^48^. BM macrophages (BMM) were obtained by culturing BM cells for 5 days in culture media containing a 1:25 dilution of supernatant from CMG 14-12 cells (CMG 14-12), as a source of macrophage colony-stimulating factor (M-CSF) as previously described^49^ or with recombinant M-CSF (BioLegend). To induce OC differentiation, non-adherent cells were removed by vigorous washes with PBS, and adherent BMM were detached with Trypsin-EDTA and plated at 0.5–1 × 10^4^ cells/well in a 96-well plate in culture media containing a 1:50 dilution of CMG. To induce OC differentiation, BMM were treated with 25–50 ng/ml receptor activator of NF-kB ligand (RANKL) on the next day after plating (D0). Bone particles or HA crystals were added on day 2 (D2) of the cultures, which were fed every 2 days afterwards as previously described^48^ and maintained at 37 °C in a humidified atmosphere of 5% CO_2_ in air.

For co-culture experiments, bone marrow stromal cells (BMSC) were obtained by culturing BM cells in α-MEM containing 20% fetal bovine serum (Invitrogen) as previously described^50^. Upon confluence, non-adherent cells were removed by vigorous washes with PBS, and adherent BMSC were detached with trypsin-EDTA. Co-cultures were prepared by plating BMSC and BMM at a 1:1 ratio (4.5–6 × 10^4^ total cells/well in 96-well plate) in medium containing 10 nM 1,25(OH)_2_D_3_ and 100 nM dexamethasone as previously described^50^. BP or HA crystals were added 2 days after the initiation of the co-cultures.

### TRAP assay

Cytochemical staining for TRAP was used to identify OC as previously described^48^. Briefly, cells in a 96-well plate were fixed with 3.7% formaldehyde and 0.1% Triton X-100 for 10 min at room temperature. Cells were rinsed with water and incubated with the TRAP staining solution (Sigma leukocyte acid phosphatase kit) at room temperature for 30 minutes. Multinucleated TRAP-positive cells with ≥3 nuclei were scored as OC under light microscopy. Samples were analyzed with an inverted Nikon Eclipse T*i*-U microscope and pictures were taken by using a coupled QImaging digital camera and MetaMorph software.

### mRNA expression analysis

Total RNA was harvested from cultured OC grown in 6-well plates using PureLink RNA Mini Kit (Invitrogen). Complementary DNA was then synthesized with the High Capacity cDNA kit (AppliedBiosystems). Gene expression was analyzed using the primers listed in Supplemental Table 1 with Terra qPCR direct SYBR Green Premix (Takara, Clontech). qPCR was performed in the QuantStudio3 cycler (AppliedBiosystems) and the results were analyzed by using the ΔΔCT method normalizing against cyclophilin B.

### Analysis of caspase-1 activity by flow cytometry

Mouse BM cells were obtained as described above. Red blood cells were depleted with red blood cell lysis buffer (Roche). Cells at 0.5–1 × 10^6^/ml were incubated with the FLICA^TM^ FAM-YVAD-FMK probe (ImmunoChemistry Technology LLC, 97) for 30 minutes at 37 °C, washed and fixed in 10% buffered formalin according to the supplier’s instructions. Samples were acquired with a FACSCalibur (BD) in the FL-1 channel, followed by analysis with FlowJo software (Tree Star, Inc.).

### Microscopic detection of caspase-1 activity

BMM were plated at 5 × 10^4^ cells per well on a 96-well plate and maintained for 24 h in culture media containing a 1:10 dilution of CMG. Cells were primed with 100 ng/ml LPS for 3 hours and incubated with 15 µM nigericin (positive control) for 30 minutes or BP (1.25–5 mg/ml) or HA crystals (0.25–1 mM) for 1 hour. Cells were incubated with the FLICA^TM^ FAM-YVAD-FMK probe as recommended by the supplier (30 minutes at 37 °C, 2 washes with PBS/FBS and fixation with 10% buffered formalin. Cells were then counterstained with Fluoro-gel II containing DAPI (Electron Microscopy Sciences). Images were taken with a DP70 Olympus Digital Camera coupled to an IX51Olympus inverted microscope, captured with QCapture Pro software and analyzed with ImageJ software.

### Western blot analysis

Cell extracts were prepared by lysing cells cultured in 6-well plates with RIPA buffer (50 mM Tris, 150 mM NaCl, 1 mM EDTA, 0.5% NaDOAc, 0.1% SDS, and 1.0%NP-40) plus phosphatase and protease inhibitor cocktails (Xpert P3200-001 and P3100-001, respectively, GenDEPOT). Protein concentrations were determined by the Bio-Rad method (DC Protein Assay), and equal amounts of proteins were subjected to SDS-PAGE gels (8–15%). Proteins were transferred onto PVDF membranes and incubated with primary antibody overnight at 4 °C, followed by a 1 h incubation with secondary goat anti-mouse or goat anti-rabbit Alexa-Fluor 680 (Molecular Probes), or donkey anti-goat 780 (LI-COR Biosciences). Primary antibodies include NLRP3 antibody (Adipogen), phospho-p65 antibody, IκB antibody, total p65 antibody and phospho-p38 antibody (Cell Signaling Technology), total p38 antibody and β-actin antibody (Santa Cruz Biotechnology). The results were visualized using Li-Cor Odyssey Infrared Imaging System (LI-COR Biosciences).

### Immunoassays

IL-1β levels in bone marrow or cell culture supernatants were determined by ELISA (eBioscience) as previously described^27, 28^.

### Statistical analysis

Statistical significance was assessed by 2-way ANOVA, unless otherwise stated, followed by an appropriate multiple comparison test (Sidak’s, Dunnett’s, Fisher’s LSD or Tukey’s) using the GraphPad Prism 6 software.

## Electronic supplementary material

Supplementary information
