# Supplementary material for: Bone matrix components activate the NLRP3 inflammasome and promote osteoclast differentiation
Source: Sci Rep. 2017 Jul 26;7:6630. doi: 10.1038/s41598-017-07014-0 (PMC5529467; doi:10.1038/s41598-017-07014-0)
Supplement: Supplementary file 1 — Supplementary information [file 41598_2017_7014_MOESM1_ESM.pdf]

Bone matrix components activate the NLRP3 inflammasome and promote osteoclast differentiation

Yael Alippe[1], Chun Wang[1], Biancamaria Ricci[2], Jianqiu Xiao[1], Chao Qu[1], Wei Zou[3], Deborah V Novack[1], Yousef Abu-Amer[2], Roberto Civitelli[1], Gabriel Mbalaviele[1*].

[1]Division of Bone and Mineral Diseases, [2]Department of Orthopaedic Surgery, [3]Department of Pathology and Immunology, Washington University School of Medicine, St. Louis, MO 63110

Supplementary information

Figure S1. Bone matrix components stimulate the expression of some inflammasome constituents. BMM from WT or *Nlrp3*$^{-/-}$ mice were incubated with 50 ng/ml RANKL in the presence of CMG as a source of M-CSF. Cells pre-treated with RANKL for 2 days (D2) were exposed to bone particles (BP, 125 µg/ml) or PBS for 24 hours or 72 hours (h). Cells were harvested on D0 (the first day of RANKL stimulation), D2, D3 or D5 and mRNA expression was assessed. Data were expressed as means ± SD, and are representative of at least 3 independent experiments. **(d)** ****p<0.0001; **(e)** *p=0.0412 (D3 vs. D3 BP 24 h), *p=0.0116 (D5 vs. D5 BP 24 h). **(f)** Full blot corresponding to figure **1a**. Arrows indicate cropped lanes. HA= hydroxyapatite.

Figure S2. Bone particles stimulate OC differentiation. BMM from WT or *Nlrp3*$^{-/-}$ mice were incubated with 1:50 dilution of CMG as a source of M-CSF **(a)**, 10 ng/ml recombinant M-CSF to ensure that CMG-effects are M-CSF-dependent **(b** and **c)** and 25 ng/ml RANKL for 2 days, and then treated with bone particles. Cells were stained for TRAP on day 3; data are representative of at least 2 independent experiments. Original magnification, x2 (a) or x4 (b). **(c)** OC number/well. Data are means ± SD of triplicates. ***p<0.0004; ****p<0.0001. p values in **(c)** correspond to significant differences relative to the control.

Figure S3. Hydroxyapatite crystals, but not collagen I fragments stimulate OC differentiation. BMM from WT or *Nlrp3*$^{-/-}$ mice were incubated with 25 ng/ml RANKL in the presence of CMG for 2 days, then treated with HA crystals **(a)** or non-mineralized collagen I fragments **(b)**. Cells were stained for TRAP on day 3, and OC number was counted. Data are representative of at least 2 independent experiments, and are means ± SD of triplicates. **(a)** **p=0.012, ****p<0.0001; **(b)** *p=0.0327, ****p<0.0001 (one-way ANOVA and Dunnett's multiple comparison test). p values in **(c)** correspond to significant differences relative to the control.

Figure S4. Bone particles promote OC formation in co-cultures and stimulate TNF-α expression. BMSC and BMM were obtained from WT or *Il-1r*$^{-/-}$ mice and the co-cultures were set up for various cell combinations at a 1:1 ratio. **(a)** Bone particles (BP, 30 μg/ml) were added at day 2 of the co-cultures, and TRAP staining was performed at day 4. Original magnification, x2. **(b)** *Tnf-α* mRNA expression in WT or *Nlrp3*$^{-/-}$ BMM treated 125 μg/ml BP. WT: ***p=0.0004, ****p<0.0001; *Nlrp3*$^{-/-}$: *p=0.0236, ****p<0.0001. Data are means ± SD, and are representative of at least 2 independent experiments, each run in triplicates.

Figure S5. The NLRP3 inflammasome promotes bone resorption in various states of accelerated bone turnover. (a) VivaCT analysis was performed on the femurs from 4-month old WT and *Nlrp3*$^{-/-}$ female mice before and at 8 weeks after OVX or sham surgery. BMD: ***p=0.0009; Tb.Th: **p= 0.0031. **(b)** Four-month old WT and *Nlrp3*$^{-/-}$ male mice were exposed to PTH (80 μg/kg/day) or vehicle for 2 weeks via osmotic pumps. Bones were analyzed by VivaCT. Values are means ± SD of the percentage of changes relative to baseline (before surgery or treatment) of 4-6 mice/group. **(c)** Post-mortem μCT scan was performed on the femurs from 3-month old WT and *Nlrp3*$^{-/-}$ male mice exposed to acute RANKL treatment (1 μg/kg once a day, for 2 days).

Figure S6. Zoledronate inhibits RANKL-induced bone loss. Three-month old WT female mice were given zoledronate (40 μg/kg) or PBS, once weekly, for 4 weeks, before GST-RANKL injection. Bones were analyzed by μCT **(a-c)**. Data are means ± SD of n=5 mice/group; **(b)** *p=0.0123; **(c)** *p=0.0146.

Figure S7. A model of inflammation actions in bone resorption. In conditions of accelerated bone turnover such as in hyperparathyroidism or estrogen deficiency (1), bone

degradation products (2) activate the inflammasomes in the OC lineage and perhaps in other cells too (3), leading to the release of low levels of IL-1β (low grade inflammation, 4). IL-1β acts directly on OC precursors or induces the production of osteoclastogenic factors (e.g., RANKL) by cells such as osteoblasts to promote OC formation and bone resorption (5). Other responses such as PARP1 degradation in OC lineage upon inflammasome activation also contribute to increased osteoclastogenesis (4, not depicted). In this model, the inflammasomes function as a positive feedback mechanism that perpetuates bone resorption.

Supplementary Table 1

**Primers**	**Sequence**
Cyclophilin B Fwd	AGCATACAGGTCCTGGCATC
Cyclophilin B Rev	TTCACCTTCCCAAAGACCAC
Nlrp3 Fwd	TGCTCTTCACTGCTATCAAGCCCT
Nlrp3 Rev	ACAAGCCTTTGCTCCAGACCCTAT
Nlrp1 Fwd	ATCCTGCCTGCAATCTCAGC
Nlrp1 Rev	ACATGTGGCTTCCATGTGCT
Nlrc4 Fwd	CCGCCCGAAAGATCATCCAT
Nlrc4 Rev	CAGGTCTTCTTCTGTGACCTGA
Aim 2 Fwd	AAATGCTGTTGTTGACCGGC
Aim 2 Rev	GAGTGTGCTCCTGGCAATCT
Asc Fwd	AACTGCGAGAAGGCTATGGG
Asc Rev	TGAGCTCCAAGCCATACGAC
Caspase 1 Fwd	GGACCCTCAAGTTTTGCCCT
Caspase 1 Rev	AGACGTGTACGAGTGGTTGT
Il-1β Fwd	GTGCAAGTGTCTGAAGCAGC
Il-1β Rev	CAAAGGTTTGGAAGCAGCCC
Il-18 Fwd	ACAGGCCTGACATCTTCTGC
Il-18 Rev	ATTGTTCCTGGGCCAAGAGG
Tnf-α Fwd	CTGTAGCCCACGTCGTAG
Tnf-α Rev	TTGAGATCCATGCCGTTG

Fig. S1

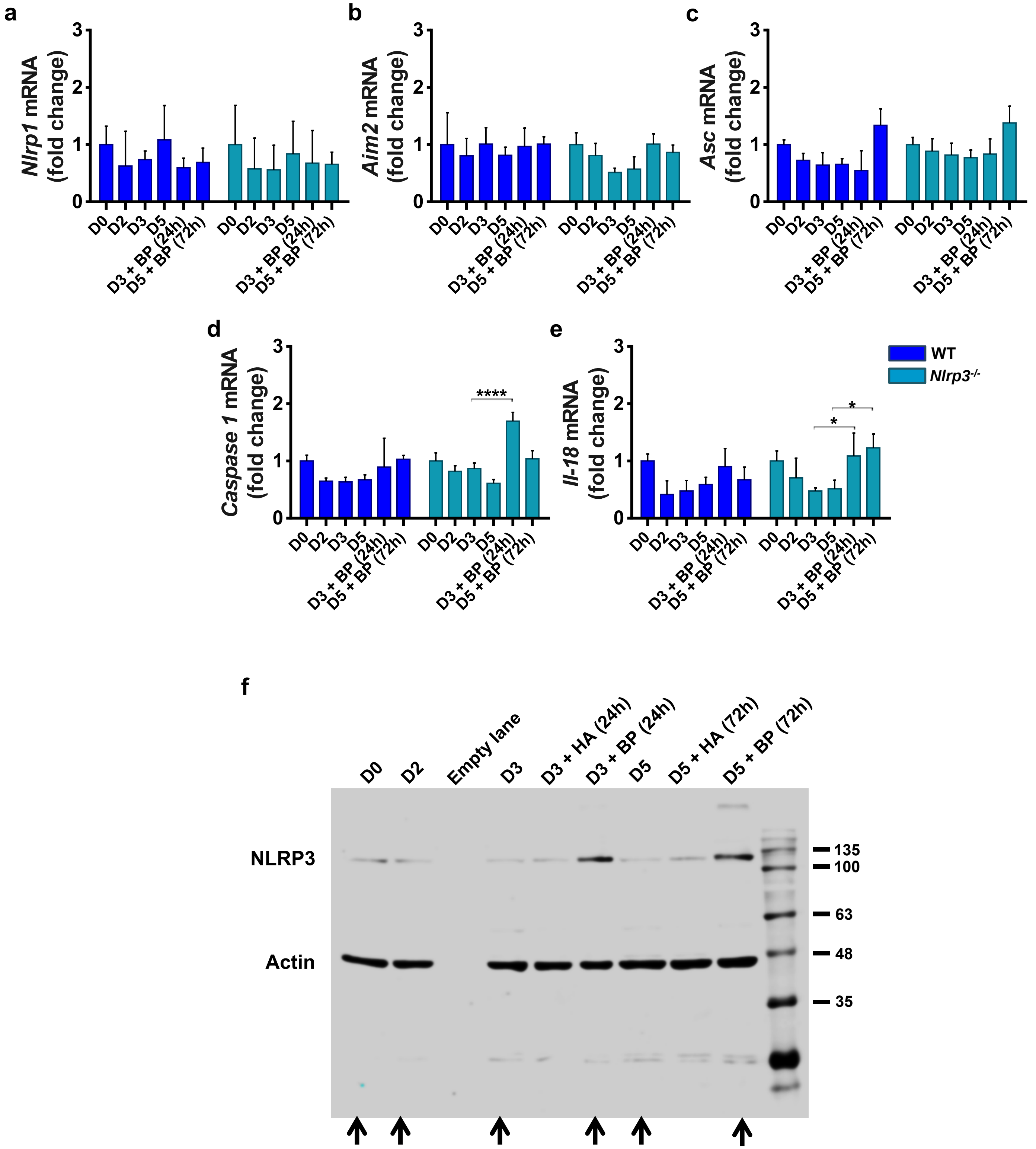
a
b
c
d
e
f
Nlrp1 mRNA (fold change)
Aim2 mRNA (fold change)
Asc mRNA (fold change)
Caspase 1 mRNA (fold change)
Il-18 mRNA (fold change)
0
1
2
3
D0
D2
D3
D5
D3 + BP (24h)
D5 + BP (72h)
****
*
*
WT
Nlrp3-/-
D0
D2
Empty lane
D3
D3 + HA (24h)
D3 + BP (24h)
D5
D5 + HA (72h)
D5 + BP (72h)
NLRP3
Actin
135
100
63
48
35

Fig. S2

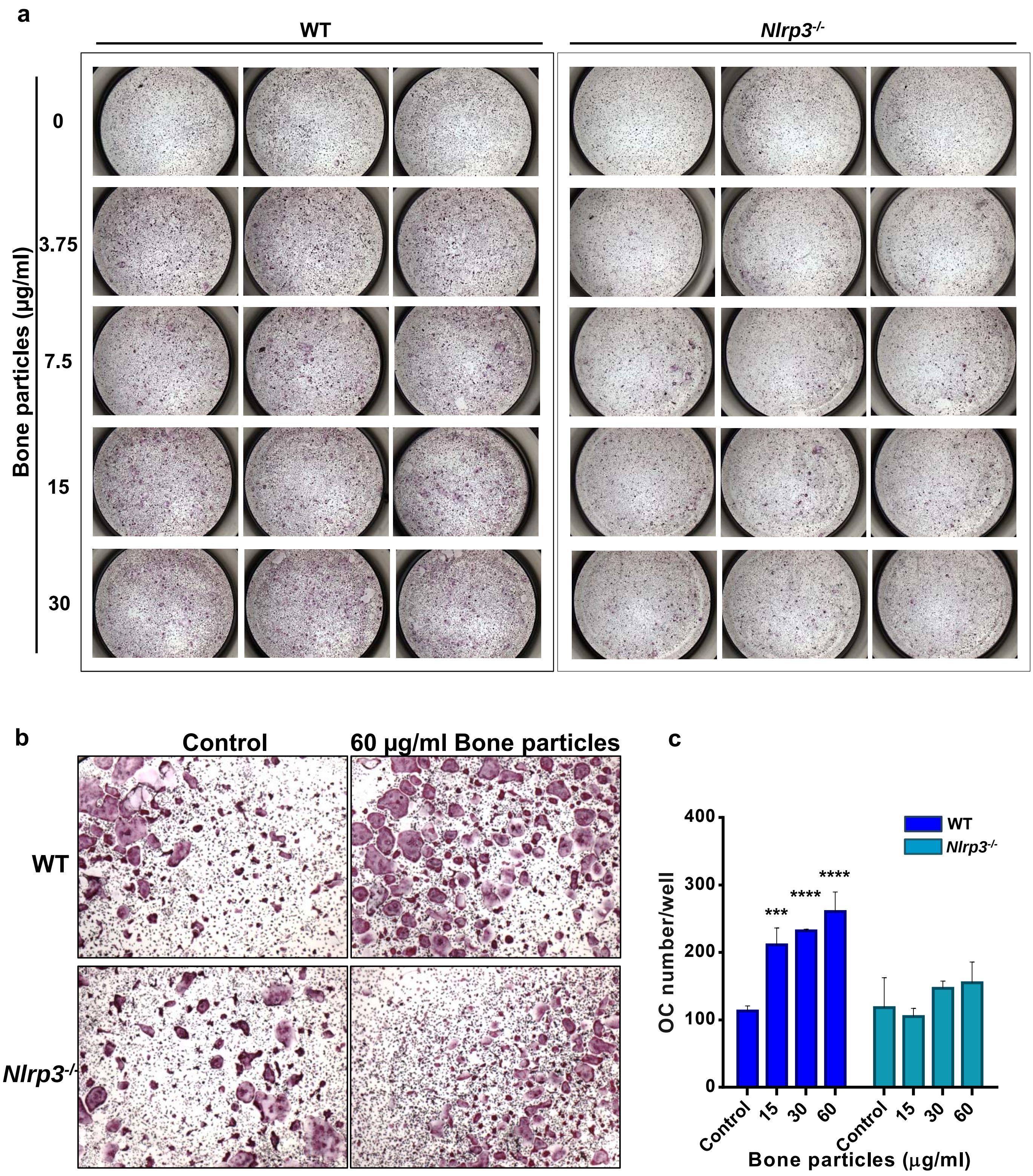
a
WT
Nlrp3-/-
Bone particles (µg/ml)
0
3.75
7.5
15
30
b
Control
60 µg/ml Bone particles
WT
Nlrp3-/-
c
OC number/well
400
300
200
100
0
***
****
****
WT
Nlrp3-/-
Control
15
30
60
Control
15
30
60
Bone particles (µg/ml)

Fig. S3

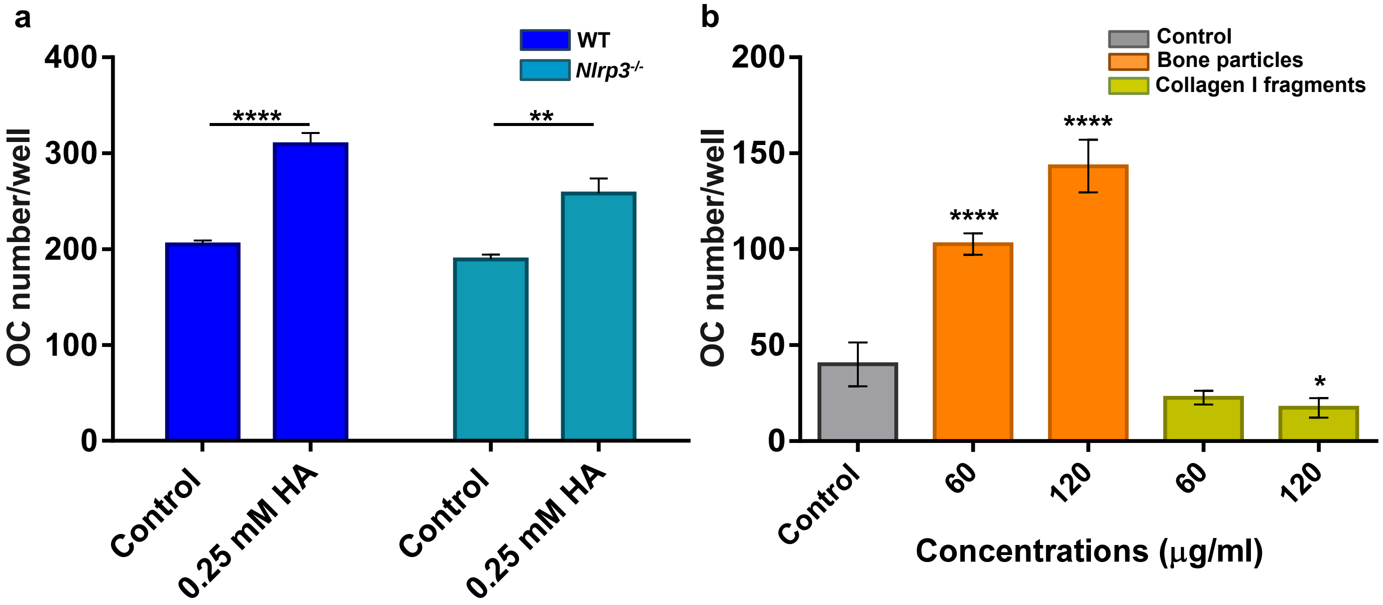
a
WT
Nlrp3-/-
OC number/well
400
300
200
100
0
****
**
Control
0.25 mM HA
Control
0.25 mM HA
b
Control
Bone particles
Collagen I fragments
OC number/well
200
150
100
50
0
****
****
*
Control
60
120
60
120
Concentrations (μg/ml)

Fig. S4

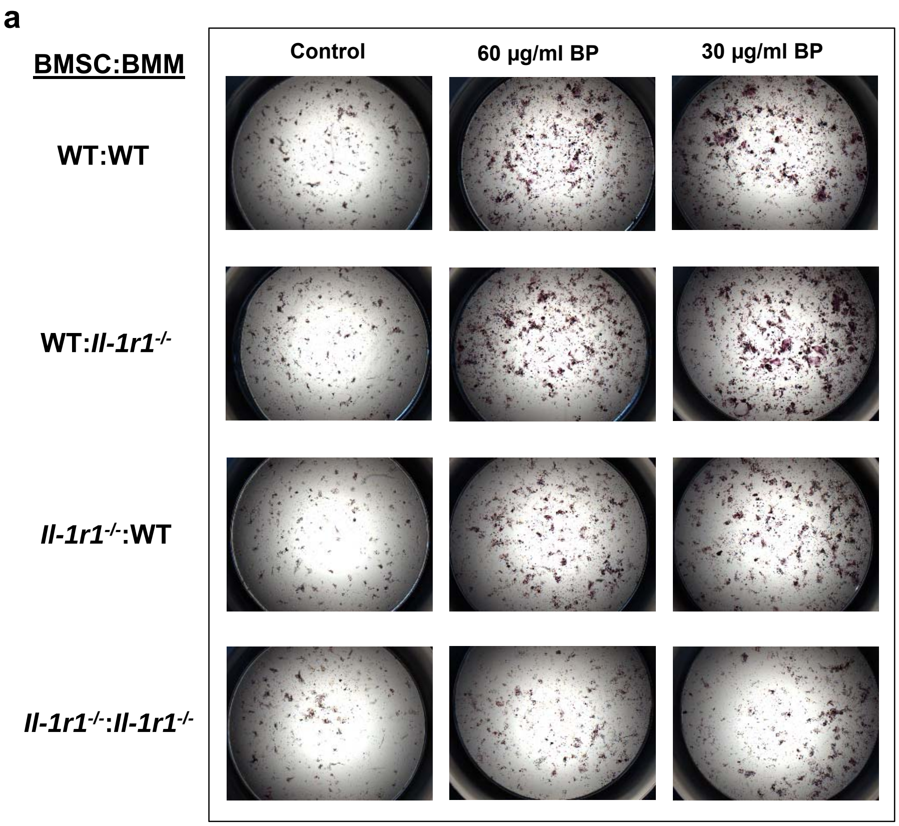
a
BMSC:BMM
Control
60 μg/ml BP
30 μg/ml BP
WT:WT
WT:Il-1r1-/-
Il-1r1-/-:WT
Il-1r1-/-:Il-1r1-/-

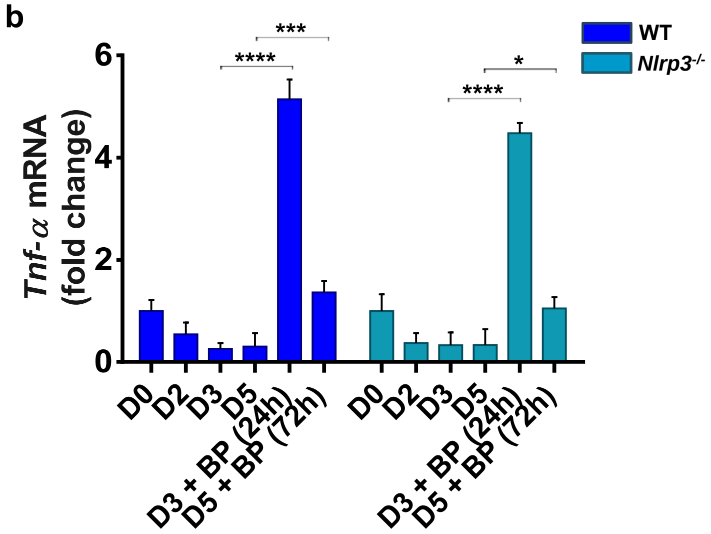
b
Tnf-α mRNA (fold change)
0
2
4
6
WT
Nlrp3-/-
****
***
****
*
D0
D2
D3
D5
D3 + BP (24h)
D5 + BP (72h)
D0
D2
D3
D5
D3 + BP (24h)
D5 + BP (72h)

Fig. S5

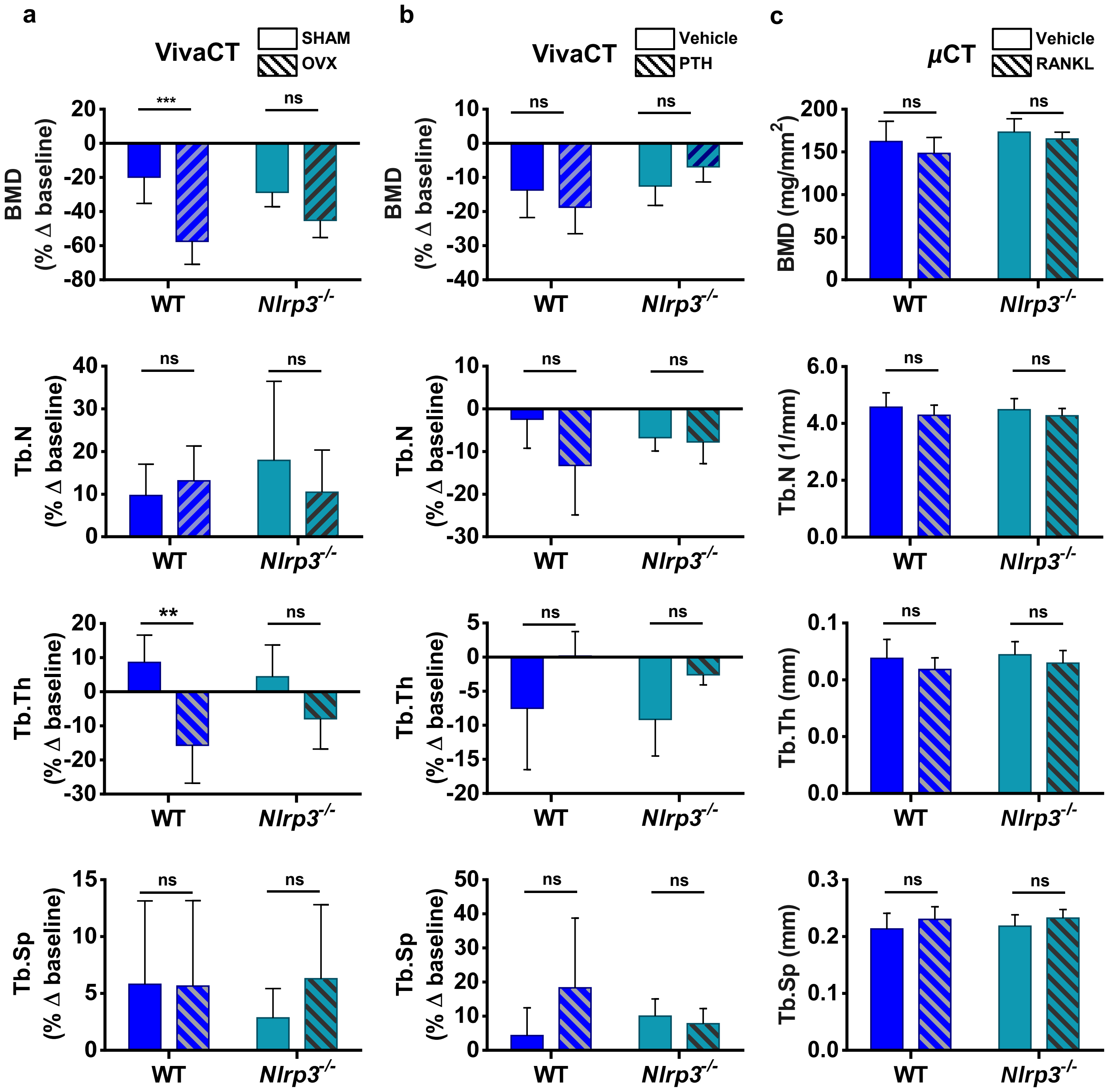
a
VivaCT
SHAM
OVX
b
VivaCT
Vehicle
PTH
c
μCT
Vehicle
RANKL
BMD (% Δ baseline)
Tb.N (% Δ baseline)
Tb.Th (% Δ baseline)
Tb.Sp (% Δ baseline)
BMD (mg/mm2)
Tb.N (1/mm)
Tb.Th (mm)
Tb.Sp (mm)
***
**
ns
WT
Nlrp3-/-

Fig. S6

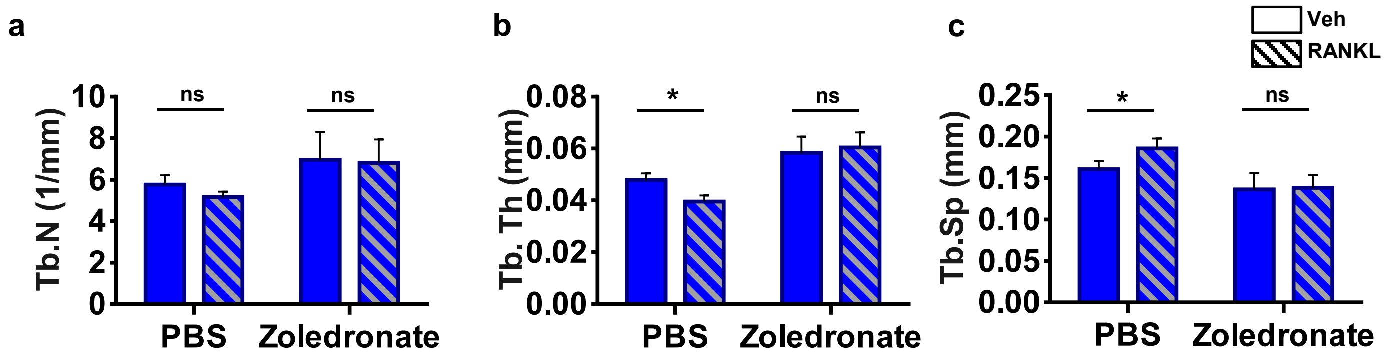

a
b
c
Veh
RANKL
Tb.N (1/mm)
10
8
6
4
2
0
ns
ns
PBS
Zoledronate
Tb. Th (mm)
0.08
0.06
0.04
0.02
0.00
*
ns
PBS
Zoledronate
Tb.Sp (mm)
0.25
0.20
0.15
0.10
0.05
0.00
*
ns
PBS
Zoledronate

Fig. S7

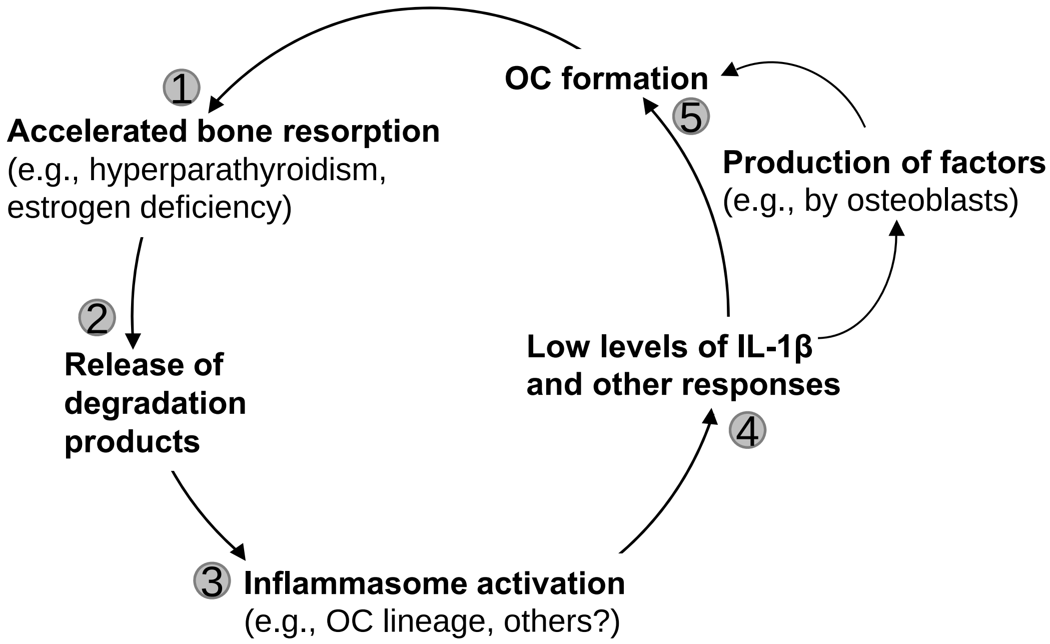

1
Accelerated bone resorption
(e.g., hyperparathyroidism,
estrogen deficiency)
2
Release of
degradation
products
3
Inflammasome activation
(e.g., OC lineage, others?)
4
Low levels of IL-1β
and other responses
5
OC formation
Production of factors
(e.g., by osteoblasts)
